# Sleep Endoscopy in the Evaluation of Pediatric Obstructive Sleep Apnea

**DOI:** 10.1155/2012/576719

**Published:** 2012-02-15

**Authors:** Aaron C. Lin, Peter J. Koltai

**Affiliations:** ^1^Division of Pediatric Otolaryngology, Children's Hospital of Los Angeles, USC Keck School of Medicine, Los Angeles, CA 90027, USA; ^2^Division of Pediatric Otolaryngology, Lucile Packard Children's Hospital, Stanford University School of Medicine, Stanford, CA 94304, USA

## Abstract

Pediatric obstructive sleep apnea (OSA) is not always resolved or improved with adenotonsillectomy. Persistent or complex cases of pediatric OSA may be due to sites of obstruction in the airway other than the tonsils and adenoids. Identifying these areas in the past has been problematic, and therefore, therapy for OSA in children who have failed adenotonsillectomy has often been unsatisfactory. Sleep endoscopy is a technique that can enable the surgeon to determine the level of obstruction in a sleeping child with OSA. With this knowledge, site-specific surgical therapy for persistent and complex pediatric OSA may be possible.

## 1. Introduction

Obstructive sleep apnea (OSA) has been estimated to affect 1–4% of all children in the United States [1] and is linked to a number of health-related issues and behavioral problems such as daytime sleepiness, enuresis, cardiovascular problems, poor growth, hyperactivity, academic difficulties, and attention issues [2, 3]. Pediatric OSA is characterized by recurrent obstruction of the airway resulting in snoring, gasping, and apneic events, and OSA in children has been recognized as a significant disorder warranting evaluation and management. In many cases, adenotonsillar hypertrophy is the primary cause of OSA in children. Therefore, tonsillectomy and adenoidectomy (T&A) is the preferred surgical method to treat OSA in most pediatric patients.

With the increased use of polysomnography (PSG), studies have shown that 15–20% of children will have persistent OSA with postoperative symptoms of obstruction [4, 5]. Children with comorbidities such as obesity, Down syndrome, and craniofacial abnormalities may have obstruction unrelated to the tonsils and adenoids; these patients are at even higher risk for persistent apnea after T&A. In addition, healthy children with OSA may have small tonsils and adenoids that are unlikely to be completely responsible for their obstruction [6]. There are a variety of causes for persistent and complex cases of OSA; these include obesity, hypotonia, craniofacial disproportion, lingual tonsillar hypertrophy, and occult laryngomalacia [7–10]. One solution to these difficult cases is the use of continuous positive airway pressure (CPAP). CPAP is generally effective in managing persistent OSA after adenotonsillectomy or complex OSA unrelated to the tonsils and adenoids, and its use is widely adopted in adults. Unfortunately, the use of CPAP in children can be difficult, and many children do not tolerate it [11]. Tracheotomy may be necessary in some severe cases of OSA.

The evaluation of children suspected of having complex OSA or persistent OSA after adenotonsillectomy should include polysomnography to confirm obstruction during sleep. Once OSA is diagnosed, identifying the site of airway obstruction during sleep may allow further interventions beyond tonsillectomy and adenoidectomy and CPAP. Flexible fiberoptic laryngoscopy is a useful tool that can be performed in the office to look for any potential levels of airway obstruction. Adenoid regrowth, lingual tonsillar hypertrophy, tongue base prolapsed, or laryngeal causes of obstruction can be seen with office fiberoptic laryngoscopy. However, this evaluation is limited by the fact that patients are awake and upright when examined; potential sites of obstruction may be missed.

Sleep endoscopy has been previously described in the adult and pediatric populations for the purpose of evaluating the dynamic airway in the supine position during a sleep-like state [6, 12, 13]. It is an increasingly useful tool in the evaluation of children with persistent OSA after initial therapy, children with OSA without tonsil or adenoid hypertrophy, or children with significant craniofacial anomalies.

## 2. Method

Sleep endoscopy is performed in the operating room under general anesthesia and is performed in conjunction with direct laryngoscopy and bronchoscopy. The child is given inhalational anesthesia by mask. The IV is then placed, and anesthesia is then maintained with an infusion of dexmedetomidine at 1-2 mcg/kg/hr without a loading dose, as well as with a concurrent ketamine bolus of 1 mg/kg as our primary anesthetic cocktail. In the past, a propofol infusion was used to maintain anesthesia. However, we have found that there is less muscular relaxation and a more sustained respiratory effort with this current technique. We also vasoconstrict and anesthetize the nose with a half and half mixture of oxymetazoline and 1% xylocaine delivered on a 1 cm × 4 cm cottonoid pledget. Spontaneous respiration is supported by oxygen (2L/min) delivered via nasal canula. The child should be in the supine position without a shoulder roll, mimicking the position of natural sleep as much as possible.

Once a rhythmic pattern of respiration is established, a flexible fiberoptic laryngoscope is passed directly into the child's nose, passing posteriorly toward the nasopharynx. For visualization and documentation, a digital video camera is used with the endoscope.

**Figure 1:**
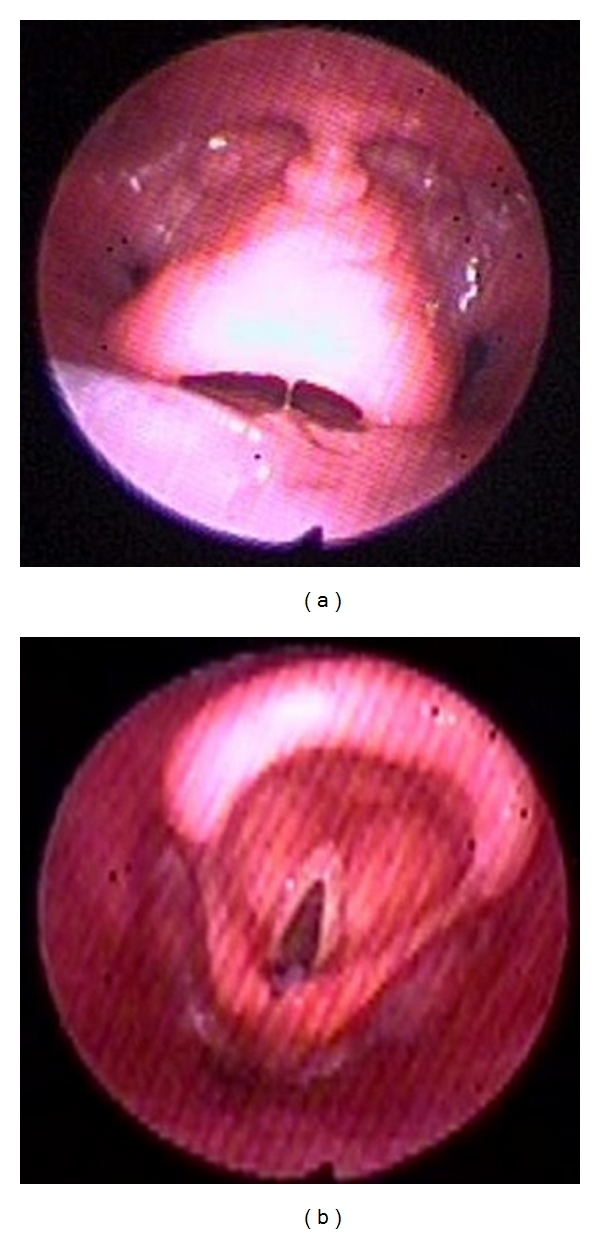
(a) Posteriorly displaced epiglottis secondary to tongue base collapse. (b) Dramatic improvement of the airway upon lifting the tongue base anteriorly using jaw thrust.

At the nasopharynx, the adenoids are examined as a potential site of obstruction. The position of the palate and uvula in relation to the posterior pharyngeal wall can be seen. The scope is then passed into the oropharynx where the tongue base, lingual tonsils, and pharyngeal tonsils (if still present) are examined. The position of the base of tongue, vallecula, and epiglottis in relation to the posterior pharyngeal wall is noted. In some cases, the tongue base can be seen collapsed against the posterior pharyngeal wall; visualizing the improvement in airway patency by lifting the tongue base with jaw thrust can be quite dramatic (Figures 1(a) and 1(b)). The dynamics of lateral pharyngeal wall motion can be seen. The scope is then passed under the epiglottis where the dynamics of the supraglottic soft tissues, as well as the motion of the vocal cords, are observed. At the completion of the sleep endoscopy, the scope is removed. Direct laryngoscopy and bronchoscopy can then be performed to complete the airway evaluation.

**Figure 2:**
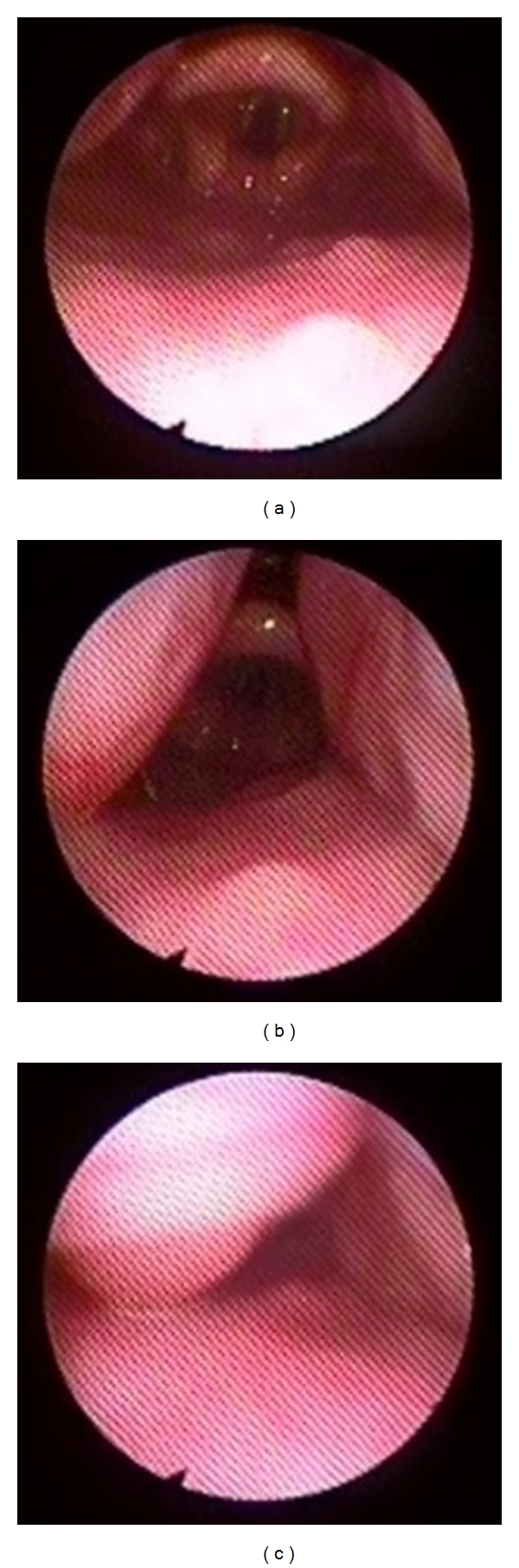
(a) Supraglottic larynx with the pharyngeal tonsils seen laterally. (b) Rapid collapse of the pharyngeal tonsils medially on inspiration. (c) Complete obstruction of the airway by the pharyngeal tonsils on inspiration. On the expiration, the tonsils returned to their normal positions as in Figure 2(a).

During sleep endoscopy, dynamic airway obstruction potentially can be observed at several levels. In the nasopharynx, the normal velopharyngeal opening should remain patent during inspiration and expiration. However, adenoid hypertrophy, adenoid regrowth, and midfacial hypoplasia can obstruct this opening and reduce nasal airflow. In the oropharynx, the airway can be narrowed by the lateral pharyngeal wall and tonsils. This space collapses and expands during respiration but normally should not result in obstruction. Not surprisingly, tonsillar hypertrophy can significantly narrow and obstruct this space (Figures 2(a), 2(b), and 2(c)). Collapse of the lateral pharyngeal walls can occur during inspiration in children with hypotonia.

**Figure 3:**
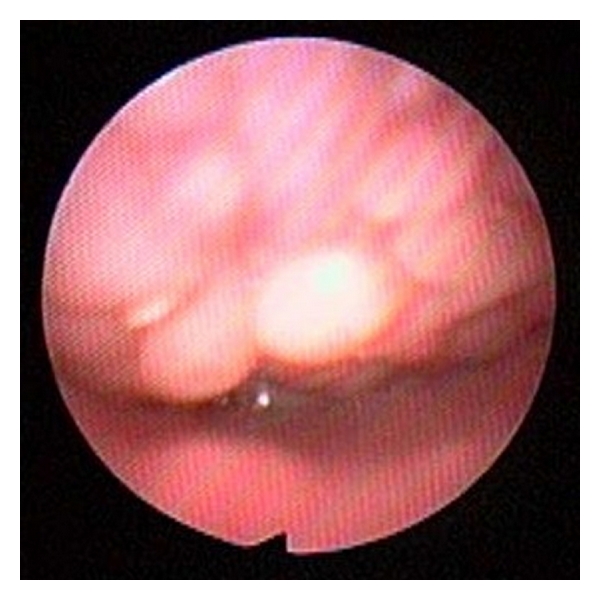
Tongue base collapsed against the posterior pharyngeal wall. The tip of the epiglottis is barely seen.

**Figure 4:**
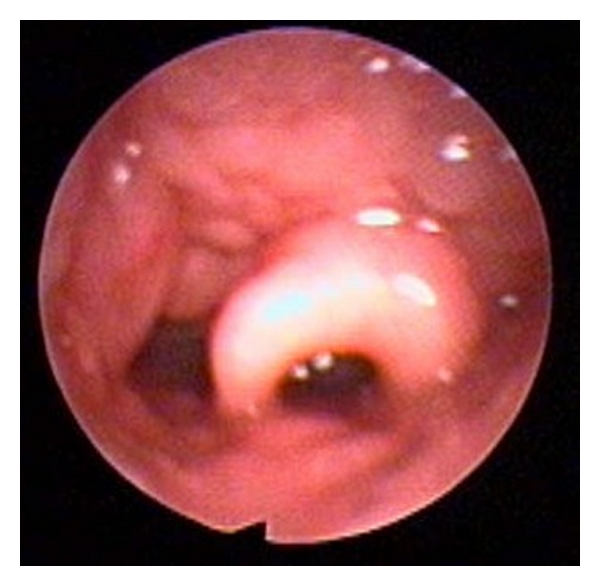
Lymphoid tissue of the lingual tonsils displacing the epiglottis posteriorly.

The tongue base should not rest on the posterior pharyngeal wall or displace the epiglottis posteriorly, allowing air to flow under and around the edges of the epiglottis. Again, hypotonia can result in collapse and obstruction at the tongue base, displacing the epiglottis against the posterior pharyngeal wall (Figure 3). Similarly, lingual tonsillar hypertrophy can crowd the entire vallecula with lymphoid tissue and displace the epiglottis against the posterior pharyngeal wall during inspiration (Figure 4). This is also seen in children with micrognathia or retrognathia where the tongue base is displaced posteriorly by the underdeveloped mandible.

**Figure 5:**
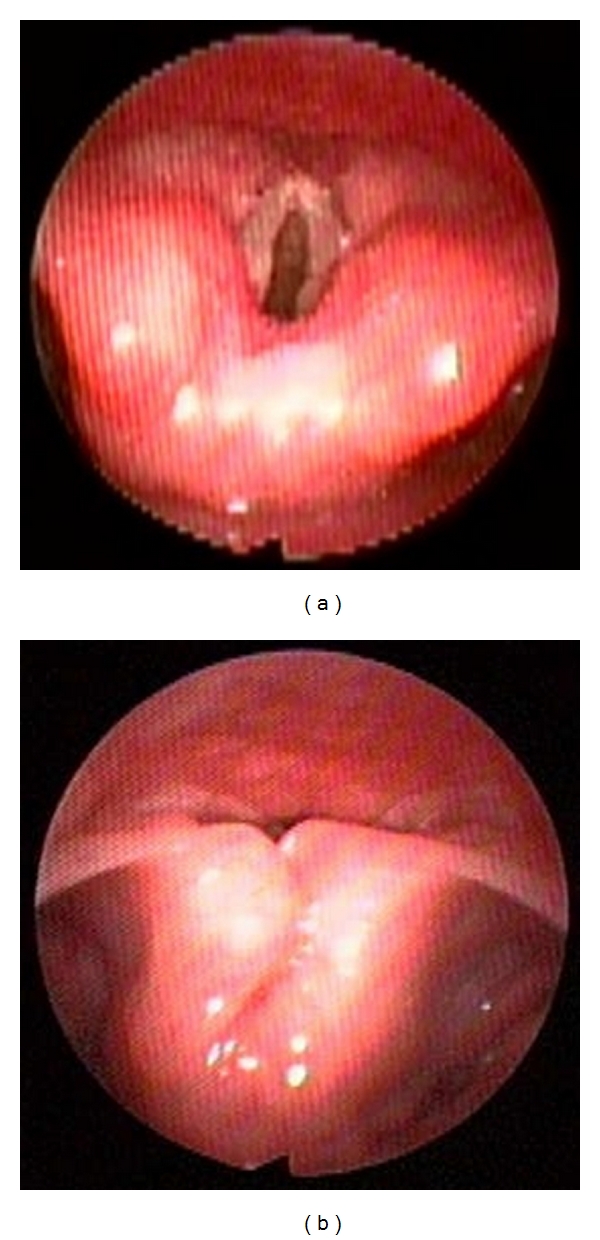
(a) Supraglottis of an older child with occult laryngomalacia prior to inspiration. (b) Upon inspiration, the redundant arytenoid mucosa prolapses into the laryngeal introitus causing obstruction.

If not displaced posteriorly by the tongue base, the normal supraglottis should remain patent during inspiration and expiration. In infants, airway obstruction may result from laryngomalacia characterized by floppy arytenoid mucosa and short aryepiglottic folds; this is present both while awake and during sleep. In some older children with persistent OSA, dynamics similar to laryngomalacia can be seen where excessive mucosal folds above the arytenoids prolapse into the glottis on inspiration. However, as opposed to infantile laryngomalacia, this form of obstruction only manifests during sleep; we call this “occult” laryngomalacia (Figures 5(a) and 5(b)).

## 3. Discussion

Sleep endoscopy was first described in 1991 by Croft and Pringle [12]. Since then, many studies have shown sleep endoscopy to be a safe and useful tool in the evaluation of the upper airway obstruction [14–16]. The findings in sleep endoscopy can assist in the management of persistent or difficult cases of pediatric OSA. Factors other than adenotonsillar hypertrophy which are known to contribute to airway obstruction during sleep include craniofacial disproportion such as midface hypoplasia or micrognathia, hypotonia, obesity resulting in oropharyngeal soft-tissue redundancy, laryngomalacia, and lingual tonsillar hypertrophy. Historically, the exact site or sites of obstruction have been difficult to evaluate and identify in the pediatric population. The use of flexible fiberoptic laryngoscopy in the office as well as rigid laryngoscopy and bronchoscopy in the operating room has aided the workup of persistent and complex pediatric OSA. However, sleep endoscopy has the advantage of visualizing dynamic collapse and obstruction of the airway during a sleep-like state.

A number of studies have examined the utility of sleep endoscopy in the management of complex and persistent OSA. Various scoring schemes have been proposed to grade and classify obstruction seen during sleep endoscopy [14–17]. Yet the ultimate goal has been to identify the site of obstruction in order to direct further interventions. Simple site-specific identification of obstructing structures (e.g., lingual tonsils or arytenoids) is likely to be most useful as any potential surgical procedures (e.g., lingual tonsillectomy or supraglottoplasty) can be directed towards these structures to relieve obstruction [18].

**Figure 6:**
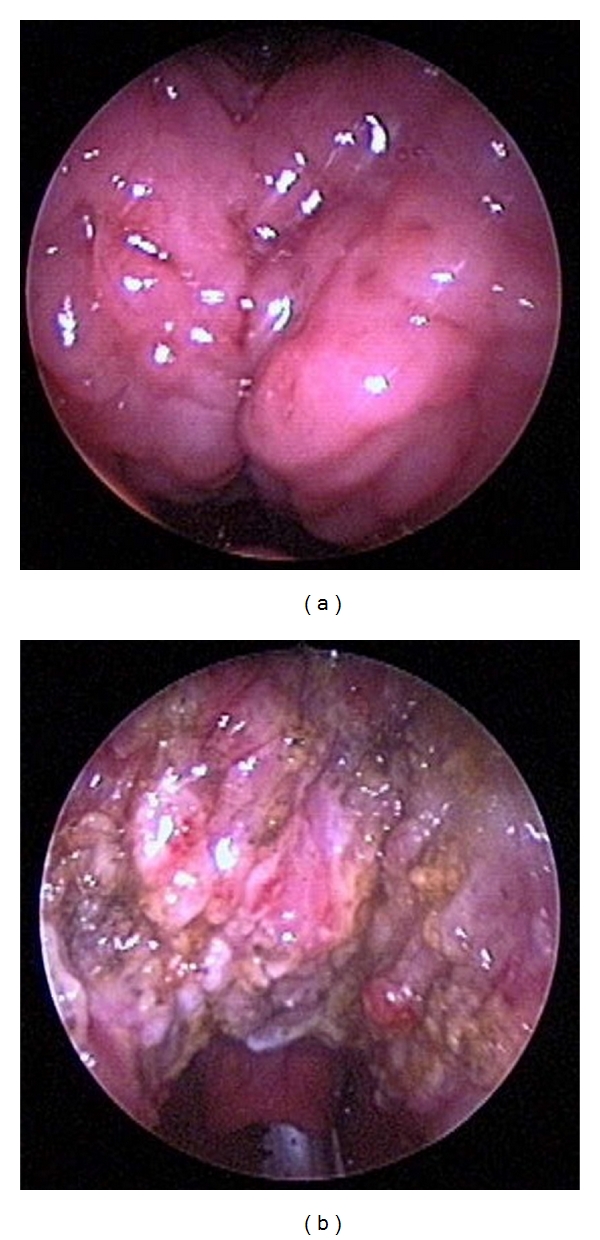
(a) Lingual tonsils prior to endoscopic-assisted coblation lingual tonsillectomy. (b) After lingual tonsillectomy, the epiglottis can now be seen.

Lingual tonsillectomy is one example. Lingual tonsillar hypertrophy has been well described in the anesthesia literature as a potential area of airway obstruction [7–10]. The lingual tonsils and the base of tongue are particularly concerning in Down syndrome with macroglossia and glossoptosis, although healthy children may have isolated lingual tonsillar hypertrophy as well [19, 20]. Using sleep endoscopy, Lin and Koltai conducted a study in which 26 children with persistent OSA were found to have lingual tonsillar hypertrophy. They reported a significant improvement in symptoms and polysomnogram scores after performing endoscopic-assisted coblation lingual tonsillectomy [20] (Figures 6(a) and 6(b)).

Another unexpected and recently described area of obstruction in older children with OSA is at the supraglottic larynx. Laryngomalacia, normally seen in infants, can be present in older children and can cause obstruction on inspiration. However, unlike infantile laryngomalacia, occult laryngomalacia is seen only in sleep. Richter et al. reported 7 patients with a mean age of 6.3 years with OSA secondary to laryngomalacia. All were treated successfully with supraglottoplasty [21]. Similarly, Revell and Clark diagnosed 19 children with a mean age of 7.3 years with laryngomalacia and OSA. Supraglottoplasty was offered to these children as well with the majority resulting in improvement or resolution of OSA [22].

The historic criticism of sleep endoscopy was related to the use of propofol [23, 24]. This drug has been shown to cause excessive hypotonia and muscle relaxation with altered airway dynamics resulting in an inaccurate model of natural slumber. While still imperfect, the combination of dexmedetomidine and ketamine provides a better simulacrum of sleep and consequently more accurate diagnosis.

## 4. Conclusion

Sleep endoscopy is a valuable tool for the treatment of complex or persistent pediatric OSA by identifying the site of airway obstruction. This enhanced diagnostic capability provides opportunities for site-specific interventions; additional options in the management of pediatric OSA other than CPAP and adenotonsillectomy may become available.
